# A Study of Psychometric Instruments and Constructs of Work-Related Stress among Seafarers: A Qualitative Approach

**DOI:** 10.3390/ijerph20042866

**Published:** 2023-02-06

**Authors:** Siti Nazilah Mat Ali, Lucian-Ionel Cioca, Ruhiyah Sakinah Kayati, Jumadil Saputra, Muhammad Adam, Roxana Plesa, Raja Zirwatul Aida Raja Ibrahim

**Affiliations:** 1 Faculty of Business, Economics and Social Development, Universiti Malaysia Terengganu, Kuala Nerus 21030, Terengganu, Malaysia; 2 Faculty of Engineering, Lucian Blaga University of Sibiu, 550025 Sibiu, Romania; 3 Faculty of Economics and Business, Universitas Syiah Kuala, Syiah Kuala, Banda Aceh 23111, Indonesia; 4 Faculty of Sciences, University of Petrosani, 332006 Petrosani, Romania

**Keywords:** work-related stress, seafarers, systematic review, semi-structured interview, seafaring context

## Abstract

Due to unpredictable and demanding working circumstances and the significant potential for dangers and accidents, seafaring has been characterised as one of the world’s riskiest and stressful vocations that lead to physical and mental health problems. However, very few instruments measure work-related stress, particularly in a seafaring context. None of the instruments are psychometrically sound. Therefore, a valid and reliable instrument to measure seafaring work-related stress is indispensable. This study aims to review work-related stress instruments and to explore the work-related stress construct among seafarers in Malaysia. This study uses a systematic review and semi-structured interviews across two phases. In Phase 1, we conducted a systematic review of several databases: Academic Search Ultimate, Emerald Journal Premier, Journal Storage (JSTOR), ScienceDirect, Springer Link, Taylor and Francis Online, and Wiley Online Library based on Preferred Reporting Items for Systematic Review and Meta-analyses (PRISMA). In 8975 articles, only 4 (four) studies used psychological instruments and 5 (five) studies used survey questionnaires to measure work-related stress. In Phase 2, we conducted a semi-structured interview with 25 (twenty-five) seafarers, online due to COVID-19 restrictions. The semi-structured interview indicated 6 (six) themes, namely, physical stress, personal issues, social living onboard, technostress, work factors, and the effect of the COVID-19 pandemic. In conclusion, the present study has identified three psychometric instruments for measuring work-related stress among seafarers: The Psychological General Well-Being Index, Perceived Stress Scale, and Job Content Questionnaire. We also found psychometric elements in some of the instruments are questionable, such as theoretical basis, construct development, and inadequate internal consistency value. In addition, this study also found that work-related stress is a multidimensional construct that needs to be studied based on work contexts. The findings of this study can contribute to the body of knowledge of a work-related stress construct in a seafaring context and could help to inform policy makers in the maritime industry. This study suggests a psychological instrument to measure work-related stress among seafarers in future studies.

## 1. Introduction

Seafaring is an operational activity on the ocean. It has been characterised as one of the world’s riskiest occupations because of the unpredictable and demanding working conditions and the high potential for risks and accidents [1,2]. Seafaring is a profession that requires workers to be onboard ships for months, sometimes years, away from home. The work-related stress of seafarers typically differs from other professions. Their working conditions are isolated, confined and prolonged [3], and seafarers may be exposed to many possible dangers, such as accidents, injuries, and diseases [4].

With radical changes and improvements to sailing conditions, seafarers may face situations potentially affecting their psychological well-being. The Maritime Knowledge Centre [5] reported that, on average, 137 ships put in total loss claims and 700 died in accidents between 2001 and 2007. Because of that, the real causes contributing to such a phenomenon are still uncertain and continue to affect seafarers’ conditions on board. Geijerstam and Svensson [5] described how most threats result from psychological distractions affecting their mental and physical conditions. In Malaysia, the ship’s crew were found dead hanging from the side of the offshore supply ship near Kemaman Port, Chukai, because they suffered from depression [6].

The stress in this profession could be physical, such as noise, weather, type of ship, and location of the seafarer. It can be associated with personal factors such as character, marital status, experience, and awareness. Potential social contributors to stress were poor support, interaction with people, and other social activities [7,8,9]. Work-related matters are also stressful, such as workload, long working hours, treatment wages, and emotional wellness [10]. All these forms of stress were identified in Iversen [1], Nurcholis and Qurniawati [11], Oldenburg and Jensen [12], Haque and Aston [13], Oldenburg and Jensen [14], Slišković [15], Carotenuto et al. [16], Rengamani and Murugan [17], Olderburg et al. [18], and Jeżewska et al. [19].

The different types of stress emphasise that seafaring is associated with numerous mental, psychosocial, and physical stressors, including fatigue, sleep deprivation, authoritative leadership, and heavy mental load. The physical workload is long work hours, loneliness, and a multinational crew [4,17,18,20,21,22]. Stressful conditions lead to unhealthy lifestyles and could lead to psychological disorders such as depression and anxiety. Many studies on stress among seafarers have found that most of the stress factors among seafarers are work-related matters [12,13,23,24,25]. Previous studies highlighted inadequate training in specific tasks, lack of control and influence, and an uncaring work environment [26]. Additionally, seafarers’ approaches to overcoming work-related stress, including intervention strategies, policies, and management practices, were discussed.

Studies have suggested numerous approaches to stress intervention, such as web counselling, psychoeducation, and web support [7,15,16,27]. However, planning and intervention should come with effective strategies. Carotenuto et al. [16] suggested identifying and providing effective intervention strategies to improve seafarers’ stressful conditions. In other words, effective intervention is not possible without properly identifying the causes of stress among seafarers. Briner and Reynolds [28] stated that good work-related stress should be measured accurately for effective intervention. A good measurement will help validate the causes of work-related stress among seafarers. Thus, it is necessary to investigate how work stress among seafarers are measured the existing studies. Additionally, Abbas et al. [29] have reviewed the stress instruments and found that the existing instruments are very general, and some do not fit with specific work settings. In the 21st century, work conditions and situations have changed. Carotenuto et al. [16] added that specific measures or instruments for seafarers’ work-related stress are needed to capture the real context of the seafarers. Besides that, Olderburg and Jensen [12] said that work-stress instruments for seafarers are not available. In conjunction with the previous issue, this study aims to review the existing instruments that used in measuring work-related stress among seafarers and explores the work-related stress of seafarers. This study could offer practical contributions as it might help researchers, professionals, and practitioners in various areas such as psychology, maritime, and health services in several aspects. Firstly, to discover the real issue in seafaring context which can contribute to more understanding of work-related stress among seafarers. Secondly, to design programmes that promote healthy development and to design as well to screen the work stress of seafarers before embarking any intervention.

## 2. Materials and Methods

This qualitative study uses a systematic review and a cross-sectional study using semi-structured interviews. This study uses two phases.

### 2.1. Phase 1: A Systematic Review

This study conducts a systematic review underpinned by the structure of Arksey and O’Malley [30]. This study adopts a rigorous process of summarising evidence and findings [31]. In addition, the framework was extended and redefined. Peters et al. [32] represents the extended and redefined version of scoping and systematic review guidelines. The systematic review protocols conducted the following procedures: objective identification, search strategy development, searching, sorting, quality assessment, charting, and synthesis of results [33]. The summary of the search strategy as seen in Table 1.

#### 2.1.1. Database Search

**Figure 1:**
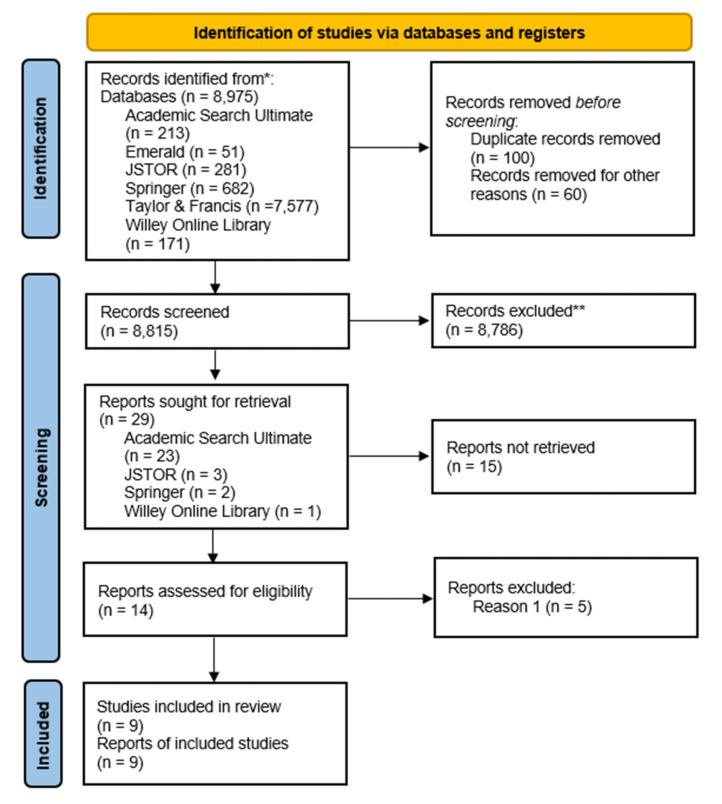
The PRISMA chart for screening, article selection, and evaluation. Note: * Consider, if feasible to do so, reporting the number of records identified from each database or register searched (rather than the total number across all databases/registers). ** If automation tools were used, indicate how many records were excluded by a human and how many were excluded by automation tools.

A total of 8975 studies were identified using the key search terms in the advanced search option of six databases: Academic Search Ultimate, Emerald Journal Premier, Journal Storage (JSTOR), Springer Link, Taylor and Francis Online, and Wiley Online Library. The key terms for searching were elaborated by combining synonyms and similar words. A synonym and text word (tw) were applied in six databases to investigate search terms to create a search strategy. All synonyms and text words (tw) with related meanings were linked with the conjunction ‘or’. The search term for work-related stress was combined with occupational or job stress and seafarers. The basic structure of key search terms was {(“Occupational Stress” or “Job Stress” or “Work-Related Stress” or “Seafarers”)}. The period of data extraction was defined during the search of the online databases from June 2020 to December 2020. The reporting of this review followed the Preferred Reporting Items for Systematic Review and Meta-Analysis Protocols (PRISMA-P). The PRISMA-P chart for screening, article selection, and evaluation as seen in Figure 1 below:

#### 2.1.2. Inclusion and Exclusion Criteria

This study included qualitative and quantitative empirical studies focused on work-related stress and seafarers. All the papers were imported from the databases into Mendeley^®^. Initially, title/abstract screening was performed during primary sorting. Later, Single Author (SA) and Multiple Author (MAB) screened all the studies using the following criteria. The criteria for inclusion and exclusion of the studies as seen in Table 2.

Table 2 displays the general inclusion criteria. It aims to restrict the number of studies because work stress is wide and may have extensive studies conducted under that scope. The inclusion criteria used were publications between 1988 and 2020 written in English, empirical studies conducted on stress among seafarers, and having to be peer-reviewed articles. Thus, dissertations and theses were not included.

#### 2.1.3. Quality Assessment and Result Synthesis

The class of the studies was evaluated using the Joanna Briggs Institute (JBI) critical appraisal tool for cross-sectional and qualitative research [34,35]. The authors (SA and MAB) evaluated the quality of the selected studies and reduced any conflict that arose during the quality assessment. All the studies surpassed the median score and were thus included. Selected studies were organised into four spreadsheets: measurement instruments, Cronbach alpha, dimensions and theoretical basis (measure). All the attributes of work-related stress were synthesised by the construct(s). Then, the sheet was summarised and pooled for systematic analysis.

### 2.2. Phase 2: A Qualitative Study Using Semi-Structured Interview

In this phase, we conducted a semi-structured interview with twenty-five (25) seafarers involving the captain, engineer, and officers. The participants were chosen using purposive sampling and collected from February until April 2021. The adequacy of the sample size is a key indicator of the research’s quality. Galvin [36] reviewed and statistically analysed the sample size for the qualitative studies. He stated that 6 (six) individuals among a probability sample of identifying a concept (theme) are greater than 99% if that concept is shared among 55% of the larger study population. Morgan et al. [37] added that the first 5 to 7 interviews produced the majority of new information in the dataset. Little further information was gained as the sample size approached 20 interviews. Participants worked onboard and at the management office in the shipping company’s fleet. Due to the COVID-19 pandemic, the interviews were conducted online via the web-conferencing tool Cisco WebEx. The interviews used digital recordings and lasted, on average, 50 min. Data on the characteristics of the participants were collected at the beginning of the interview session.

## 3. Results

### 3.1. Valid and Reliable Instruments in Measuring Work-Related Stress among Seafarers

The primary search identified 8975 articles through 6 databases: 213 articles from Academic Search Ultimate, 51 articles from Emerald journal, 281 articles from Journal Storage (JSTOR), 682 articles from Springer Link, 7577 from Taylor and Francis Online, and 171 articles from Wiley Online Library. The screening of related articles on stress among seafarers identified 29 articles, 23 from Academic Search Ultimate, 3 from JSTOR, 2 from Springer Link, and 1 from Wiley Online Library. There were 14 eligible articles based on the general inclusion criteria, and 5 articles were excluded because the Cronbach alpha did not report in the studies. Out of the 9 articles, 4 studies used psychological tests to measure stress [7,16,27,38], and 5 articles used survey questionnaires (Table 1). The psychological tests used were the Psychological General Well-Being Index (PGWBI), Perceived Stress Scale (PSS), and Job Content Questionnaire (JCQ).

The Psychological General Well-Being Index (PGWBI) was used by Carotenuto et al. [16] to measure self-perceived evaluation of stress among seafarers and has a good internal consistency. The PGWBI was developed by the National Institutes of Health (NIH) for the first large-scale survey of the physical health of the American population, known as the National Health and Nutrition Examination Survey (NHANES). This instrument measures the level of subjective psychological well-being. This instrument comprised six domains; anxiety, depression, positive well-being, self-control, general health, and vitality [39]. However, those six domains were found not specifically to measure stress. Thus, it did not represent a stress measurement, and the instrument did not report any theoretical basis.

The PSS is an instrument that Cohen, Kamarck, and Mermelstein [40] developed to measure individuals’ stress in the last month. This instrument was developed based on the Lazarus Original Transactional Model. It measures the perception of stress in one’s life [40]. Thus, the instrument was more general in measuring stress and did not specifically focus on work-related stress matters. Therefore, the instrument’s validity in measuring seafarers’ work-stress was questioned. The PSS has 3 versions: 14items, 10 items, and 4 items. The scale’s internal consistency was initially acceptable based on the previous study. Cohen and Williamson [41] reported the internal reliability for the PSS-4 (0.60), the PSS-10 (0.78), and PSS-14 (0.75). According to Tavakol and Dennick [42], the acceptable alpha threshold was above 0.70. Studies by Doyle et al. [27], and McVeigh et al. [7] among seafarers used PSS-4 and reported the internal consistency of the instrument, 0.57 and 0.55, respectively. The internal reliability of the instrument used failed to achieve the threshold level. Hence, the reliability of the scale was questionable. However, Rydstedt and Lundh [38] reported a good internal consistency (0.84) for PSS-10.

The Job Content Questionnaire (JCQ) is an instrument designed to measure scales assessing psychological demands, decision latitude, social support, physical demands, and job insecurity. This instrument was based on Job Demand Control Model [43]. Rydstat and Lundt [38] used the JCQ in their study to measure psychosocial work-stress among seafarers, comprising three domains; demands, work-related control, and work-related social support. The internal reliability of the 3 domains ranged from acceptable to good alpha Cronbach values, 0.69, 0.77, and 0.80, respectively. The instrument was found to be a work-related stress instrument. However, the instrument was questionable because the instrument was too general to fit any work context [44] and more focused on non-seafaring work contexts.

An et al. [45] and Håvold [2] used the questionnaire survey adapted from the literature. In contrast, the questionnaires used by Rengamani and Venkatraman [25] and Rengamani and Murugan [17] were constructed for their specific studies. Elo [46] reported that the questionnaire used was adapted from past studies and claimed to have been used in several epidemiological studies conducted in Finnish. All the studies reported the accepted value of internal consistency. However, these questionnaires focused more on survey purposes, did not intend to capture the work-stress construct, and lacked a theoretical basis. Table 3 presents the results of the reviewed literature.

### 3.2. Work-Related Stress Constructs among Malaysian Seafarers

The interview result indicated six themes related to seafarers’ work-stress, including physical stress, personal issues, social living on board, technostress, work factors, and the effect of the COVID-19 pandemic.

#### 3.2.1. Physical Stress

Participants reported several issues under a theme of physical stress, which include noise, weather, type of ship, and location of the seafarer. The issues are the sub-themes of the physical stress. First is the weather. The participants reported the weather affected seafarers during working onboard. Second is a type of ship in which, according to the participants, every ship has a different environment with different exposure. The different environment results in differences in stress. Additionally, the experience of working on a certain type of ship can make the seafarers feel less stressed because they used to work on the same type of ship. The third sub-theme concerned under this theme of physical stress is the location. The seafarers’ workplaces will also create different working environments and lead to different stress levels. For example, working at the port and in the middle of the ocean are two different conditions; in the middle of the ocean, they can see the ocean around them, the moon and stars at night and the sun during the day. The next sub-theme is noise on the ship caused by ship operations, such as the engine. It might cause additional stress if our resting place on the ship is near the engine room. Such conditions surely affect the seafarers and their rest quality. Lastly, confined space was a problem raised by the participant, where they needed to share small spaces with others on board. The examples of the responses mentioned in the Table 4.

#### 3.2.2. Personal Issues

There are several matters concerned under the personal factor theme. They are seafarers’ character, background, social status, passion, awareness, and experience. The first one is the character of seafarers. The character of seafarers is an important aspect to ensure they can survive in their careers; the life on the ship is tough. It is mentioned by the three participants below. One participant explained how different outgoing characters might feel more stress if they find no friends to interact with, compared to non-outgoing people. Another participant informs us that a good attitude and competency are two important elements to reduce stress while working on board. The next sub-theme is the background of the seafarers. According to the participants, the background of seafarers can have an effect, such that those from a rural area could be more resilient than those who are not, because they have experienced a tough life. 

The other participant mentioned that the seafarers who lived in their comfort zone also might face difficulties adapting to the tough life on the board. The next sub-theme under the personal issues theme is social status. The participants agreed on an example of married seafarers being more stressed than single ones. According to a participant, most married members are more likely to miss their family, thereby disturbing their work concentration, as they may worry because they cannot be there for them. The next sub-theme is passion. Passion is highlighted as an important aspect of going through a career as a seafarer. The seafarers will not enjoy it if passion is not within them. Awareness is another issue within the personal factor. There are participants who mentioned that they are unaware of their conditions and sometimes could not identify whether they are in stress. The last sub-theme is experience. Most participants associated the inexperience of the seafarers with those newly joining, as they had never experienced working onboard and they needed to adapt to life on board. The examples of the transcripts of the participants’ responses are presented in Table 5. 

#### 3.2.3. Social Living on Board

The third theme found in this study is social living onboard. The most obvious concern under this theme is interaction with people onboard. The number of people onboard also affects the stress levels of seafarers because they need friends to interact. Second, under this theme, fewer social activities contribute to seafarers’ stress. Under the theme of social living, another sub-theme that creates stress among seafarers is when they cannot escape conflict among crew members. All the transcription of responses of the sub-themes is shown in Table 6. 

#### 3.2.4. Technostress

The emergence of technology contributes to many issues that lead seafarers to stress. They include getting information through technology, which causes them more work and affects their resting time. Technology also affects concentration because sometimes information is not due to work matters, such as family problems. The next one is a skills deficit due to technostress because the technology reduces manpower (see Table 7). 

#### 3.2.5. Work Factors

Several issues highlighted under the theme of work factors include no guidance, workload, treatment of the employer, job security, working hours, and wages. The first sub-theme under this theme is ‘no guidance being given to the seafarers’. The participants’ mentioned seafarers are more likely exposed to stress when they do not get guidance from the senior officer and supervisor and need to handle most things themselves. When they failed, they were criticised for not being proactive. The next sub-theme is workload. Workload leads the seafarers to do the job out of the job scope, resulting in ineffective rest. The third sub-theme is the treatment of the employer. When the employer does not treat the seafarers properly, such as ignoring a complaint that was made, being racist, biased, and not checking on their problems, the employees might become stressed, as revealed. The responses of this theme can be referred to in Table 8.

#### 3.2.6. COVID-19 Pandemic

This issue is an important concern, as all participants raised this matter. This is the last theme found, which is the COVID-19 pandemic. The pandemic affected their career and caused them many stress-related issues. The participants’ concerns under this theme are travel restriction, no income, uncertainty, double-standard quarantine, and standard operating procedure (SOP) restrictions. The first sub-theme is travel restrictions. The seafarers address the travel restriction, as they cannot move. If they are onboard, they must stay; if they are home, they may not know when to return to work. As a result of being unable to travel, their income ceased, and they do not get paid to survive. The is the second issue highlighted. The other concern due to this pandemic is uncertainty. As highlighted by the seafarers, uncertainty is uncertainty about getting and securing a job. The next issue or the subtheme is double-standard quarantine. The participants mentioned that the number of days for quarantine differs among the seafarers. Thus, the double-standard issue is raised. The last one that caused the seafarers stress is the restrictions specified under the SOP of this COVID-19. The transcription of responses is presented in Table 9.

## 4. Discussion

Our results revealed that only three psychometric instruments were found to be used, which are the Psychological General Well-Being Index (PGWBI), the Perceived Stress Scale (PSS), and the Job Content Questionnaire (JCQ). However, based on the search process, the available studies used instruments that may have questionable elements, such as a theoretical basis and inadequate internal consistency value. The available studies’ instruments did not tap the measured construct. PGWBI instruments focus more on well-being and do not develop based on theories. Other studies used questionnaires that do not have any theoretical basis. The questionnaire was developed based on literature, constructed specifically for their study and adapted from past studies. However, those studies are still questions regarding constructing the questionnaire. Rengamani and Venkatraman [25], Rengamani and Murugan [17], and Elo [46], reported that the questionnaire is based on the list of work-related stress items without highlighting the construct development process. It may lead to psychometric issues, particularly the validity of the constructs, whether it measures what it is supposed to measure. Another study used PSS, which is a general instrument to measure perceived stress and may not be suitable for measuring work stress. Additionally, the internal consistency reported in some studies does not achieve the accepted level, but surprisingly, there were studies used to measure stress among seafarers [7,27]. JCQ may be considered an adequate instrument to measure work stress, but it is still not tapped into the seafaring context.

Six themes were generated from the interviews with the seafarers: physical stress, personal issues, social living on board, technostress, work factors, and the effect of the COVID-19 pandemic. Previous findings support these findings, except no obvious findings related to the technostress stressor. It might be more obvious during the current pandemic among seafarers as information and communication technology (ICT) becomes the main medium of interaction in all angles of human life. This study seems to acknowledge the universal stressor faced by seafarers worldwide.

The physical stress derived from the interview is the weather, noise, ship type, and seafarer’s location. These aspects have been widely mentioned in many studies such as Nurcholis and Qurniawati [11], Oldenburg and Jensen [12], Oldenburg and Jensen [14], Carotenuto et al. [16], Rengamani and Murugan [17], Olderburg et al. [18], Jeżewska, Leszczynska & Jaremin [19], and Kadhim et al. [47]. The physical aspect of stress was, for example, hot weather disturbing the concentration of work when working. Olderburg et al. [18] again mentioned that although ships nowadays are mostly air-conditioned, the weather still affects them. Seafarers also struggle with confined spaces. This physical stress also affects resting quality and job satisfaction.

The next theme is personal issues. The personal issues highlighted by the participants include seafarers’ character, background, passion, social status, awareness, and experience. This factor was a concern in Nurcholis and Qurniawati [11], Carotenuto et al. [16], Rengamani and Murugan [17], and Olderburg et al. [18]. Seafarers, such as outgoing people, are more likely to feel stress if they cannot interact with people than those who are not outgoing [48]. Working on the ship is tough, and seafarers not used to this lifestyle might take time to adapt. In addition, experience is important, as highlighted by Allen et al. [49], so that experienced people might have less stress working as seafarers compared to those who are not. According to the social status of the seafarers, single and married differ in their stress level. Those who are married mostly feel the separation from their family, as Carotenuto et al. [16] and Iversen [1] stated in their study.

The next theme is social living onboard. This matter was highlighted in McVeigh et al. [7], Carotenuto et al. [16], Iversen [1], and Jeżewska et al. [19]. Seafarers need people to interact with on board. The interaction could relieve their stress and reduce loneliness and social support. In addition, Papachristou et al. [50] stated that the social living culture on board is important for the retention of seafarers in their careers. McVeigh et al. [51] found that the decline of socialisation or social interaction on board leads to more social issues like alcohol and drugs. Besides, fewer social activities onboard could create stress. When facing conflict among themselves, they feel difficulty and stressed because they cannot escape. Slišković [15] mentioned both factors.

The fourth theme is technostress. Technostress is stress due to technology. Brivio et al. [52] defined technostress as an inability to cope due to ICT. This technostress happens among seafarers as they must acquire much information through technology, which causes them more work and affects their resting time. Studies on technostress at work agreed that although technology made life easier, it also contributes to other problems, such as increased workloads and longer working hours [52,53]. Technology also provides information faster; information not due to work matters, such as family problems, can be known although they are far away from family. It affects their work concentration because they cannot be there for their family members to help them.

Work matters is another theme derived from the study. Participants under this aspect raise some points, such as the workload, long working hours, no guidance, treatment of employer, and wages. These matters have been highlighted in Iversen [1], Oldernburg and Jensen [12], Slišković [15], Carotenuto et al. [16], and Jeżewska et al. [19]. Slišković [15] mentioned that workload and long working hours could lead to other factors such as loss of concentration, insomnia, anxiety, and headache. Iversen [1] highlighted that a high workload could lead to burnout. Rengamani & Murugan [17] explained the issue of bias and racism due to preferences that exist by management towards a particular group. This unpleasant situation of work factors is much related to the management. As mentioned by Oldernburg and Jensen [12] and McVeigh et al. [51], good management plays a huge role in creating a good working environment and it is very important to help the seafarers to cope with the challenging situations faced by seafarers and also to ensure that the stress among seafarers could be controlled.

The last theme derived from the study is the effects of the COVID-19 pandemic. This pandemic has also been an issue raised by the participants because it is an ongoing and global pandemic. Several matters are highlighted in this study: travel restriction, no income, uncertainty, double-standard quarantine, and Standard Operational Procedure (SOP) restrictions. Slišković [54] showed that this COVID-19 issue had given travel restrictions to seafarers; as with the closing of national borders, international flight tickets were cancelled. The seafarers reported having extensions in either of these two: staying at home or onboard. There is no sign-in and sign-off when working onboard. They must stay onboard for a prolonged period, which will affect their mood and emotions, and some have had their health disrupted. Slišković [54] also reported feeling worse than in prison, where they experience uncertainty when they sign off as they feel lonely and miss their family. For seafarers who must stay at home, their worry is more on the financial situation as they do not have the income to feed their families. Pauksztat et al. [55] also highlighted this issue.

Working under stress is dangerous, as it could worsen job performance and job quality, life satisfaction, and the well-being of the workers. Most of the time, work stress could threaten the feeling of enjoyment and satisfaction among workers [56,57,58]. Work stress is multidimensional; thus, it is crucial to be seen from various perspectives; every individual may have different stress factors. They know the ship as a unique place, and the workers who agree to commit to the career of working on the ship should bear with the environment and culture of limited movement and space on the board. McVeigh et al. [24] stated that seafarers work amidst a fusion of paradoxes, such as social exclusion and continuous social proximity, confinement in open spaces, and multiculturalism within the distinct organisational culture of a ship. The ship should have a good culture for the workers to enjoy and be satisfied working there.

## 5. Conclusions

In conclusion, the present study focuses on work-related stress among seafarers, particularly on the psychological perspective, which is very limited and unexplored worldwide. The main drawback in those studies is due to the questionable instruments used to measure work stress in seafaring settings. Some of the instruments lack a theoretical basis, have inadequate internal consistency, and have inadequate instruments to capture work stress in the seafaring context and its development process. Therefore, a call for a valid and reliable work-stress instrument is indispensable. Such instruments are also very important in designing the intervention and formulating policies related to work stress and wellbeing among seafarers in the maritime industry. In addition, the work settings landscape has become more complex due to the industrial revolution 4.0 and the COVID-19 pandemic. Again, the revision of the instruments and suggestions for a specific instrument for work stress among seafarers may be needed.

Besides that, this study also has identified several challenges faced by seafarers which contribute to work-related stress, such as spending time away from your loved ones, a lack of a social life or missing your buddies, and poor motivation, which can lead to a lack of interest in your work, not being able to get a good night’s sleep onboard your ship, and feeling like you cannot communicate with your fellow crew due to cultural or language differences. Additionally, the seafarers are not dealing with kids’ school issues, getting the car fixed, or dealing with all the life admin that gets thrown your way when working away from home. This study’s findings can assist seafarers in handling work-related stress by spending time, sharing special moments, and creating new memories with loved ones.

In addition, this study only uses the several databases and cannot be generalized. Thus, future research suggests considering the Scopus and Web of Science (WoS) databases. Additionally, this study did not prospectively register the study protocol in PROSPERO. Thus, future study can consider adding the study protocol (PROSPERO). Although the results of the current study cannot be generalised, it provided rich insights into the causes of work stress and new directions in the study of work stress among Malaysian seafarers, especially during the pandemic. Studying the causes of work stress among seafarers worldwide can help to offer a theoretical framework for work stress or stress reactions in seafaring settings and can also help to develop a better psychological instrument to capture the work-stress construct among seafarers. 

This study can assist seafarers in having time alone to relax and catch up with loved ones via social media, which is crucial. Still, the power of community spirit cannot be overstated. Several tips can be used for socialising when working in seafarer jobs, such as creating a chat group, heading to the gym, arranging a movie night, organising a gaming tournament, getting sporty, and learning from fellow seafarers. In addition, this study can help seafarers maintain a sense of pride in their work. The seafarers can realise how much people value seafarers’ jobs. Working as a seafarer is very important in how the world operates globally. A good night’s sleep is essential for a happier and healthier life. When someone consistently has low-quality rest, it can affect the physical, mental, and quality of life, especially working in jobs at sea (e.g., safety, etc.). This study’s findings can be helpful to seafarers in practising good sleeping habits, and it is as significant as regularly exercising, eating healthily, and not smoking.

## 6. Policy Implications

COVID-19 affects seafarers significantly. The present study contributes to identifying the stress-related instruments used by empirical studies among Malaysian seafarers and to have a better understanding and insights into stress-related issues in seafaring professions. The interview data could provide a basis for policy implications for seafarers in the future if there is a need to provide intervention strategies to manage stress among seafarers. Since stress onboard cannot be avoided due to the nature of working with the ship environment, noise, and operation of the ship, other parts of problems such as reducing long working hours and social activities should be organised to reduce stress onboard. Other issues such as job security, wages, and management should be further discussed to have more retention rates among seafarers. In addition, a policy of happy ships should be offered with a stress management programme, support groups, and other related skills training. These policy implications are important as seafarers are needed for economics and global contribution.

## Figures and Tables

**Table 1:** The summary of the search strategy.

Source(s)	Identification(s)
Academic Search Ultimate	(TITLE((“Work-stress AND Seafarers” OR “Job Stress AND Seafarers” OR “Occupational Stress AND Seafarers” OR “Seafarers”)) AND TITLE ((“Work-stress OR occupational stress OR job stress AND seafarers”))
Emerald Journal	(TITLE((“Work-stress AND Seafarers” OR “Job Stress AND Seafarers” OR “Occupational Stress AND Seafarers” OR “Seafarers”)) AND TITLE ((“Work-stress OR occupational stress OR job stress AND seafarers”))
JSTOR	(TITLE((“Work-stress AND Seafarers” OR “Job Stress AND Seafarers” OR “Occupational Stress AND Seafarers” OR “Seafarers”)) AND TITLE ((“Work-stress OR occupational stress OR job stress AND seafarers”))
Springer Link	(TITLE((“Work-stress AND Seafarers” OR “Job Stress AND Seafarers” OR “Occupational Stress AND Seafarers” OR “Seafarers”)) AND TITLE ((“Work-stress OR occupational stress OR job stress AND seafarers”))
Taylor & Francis Online	(TITLE((“Work-stress AND Seafarers” OR “Job Stress AND Seafarers” OR “Occupational Stress AND Seafarers” OR “Seafarers”)) AND TITLE ((“Work-stress OR occupational stress OR job stress AND seafarers”))
Wiley Online Library	(TITLE((“Work-stress AND Seafarers” OR “Job Stress AND Seafarers” OR “Occupational Stress AND Seafarers” OR “Seafarers”)) AND TITLE ((“Work-stress OR occupational stress OR job stress AND seafarers”))

**Table 2:** Criteria for inclusion and exclusion of the studies.

Criterion	Inclusion	Exclusion
Period	1988–2020	Outside of those years
Language	English	Non-English
Article type	Original (qualitative and quantitative)	Other than the original (any review, letter and editorial, or thesis)
Study focus	Work-related stress; Seafarers	Other than inclusion criteria
Perspective	Psychological health	Medical and experimental health

**Table 3:** Summary of reviewed literature (work-related stress among seafarers).

Author(s) (Year); Article Title	Study Design	Study Population and Sample	Dimensions	Measurement Instruments; Cronbach Alpha	Theoretical Basis (Measure)	Data Collection Method	Related Findings
McVeigh, J., MacLachlan, M., Coyle, C., & Kavanagh (2019) [7] Identifying predictors of stress and job satisfaction in a sample of merchant seafarers using structural equation modelling	Quantitative and cross-sectional design (two phases of study)	Merchant seafarers (n = 575). They are officers and ratings/crew working in the organization’s fleet on liquefied natural gas carriers, product oil tankers, and crude oil tankers, on a global basis.	No dimensions	Perceived stress scale version 4 (PSS4) The value is 0.55 and considered accepted as the same value has been found by the test developer, Cohen et al. (1985) [40]	Lazarus original transactional model	A secondary data analysis, using questionnaires administered at two time points to seafarers within a large shipping organization	Dispositional resilience and instrumental work are the important contributors to psychosocial well-being in this sample of merchant seafarers
Doyle, MacLachlan, Fraser, Stilz, Lismont, Cox & McVeigh (2016) [27] Resilience and well-being among seafarers: cross-sectional studyof crew across 51 ships	Quantitative and cross-sectional design	Seafarers from an international shipping company (n= 387) ratings, crew, officers, engineers, and catering staff that had been on board their ship between 0 and 24 weeks.	No dimensions	Perceived Stress scale version 4 (PSS4) The value is 0.57, comparable to estimates in the literature (Cohen and Williamson 1988) [41]	Lazarus original transactional model	Data from questionnaires were emailed to 53 tanker vessels in an international shipping company	Duration at sea was not related to stress among seafarers and the result found high level of resilience, longer seafaring experience and greater instrumental work support was significantly associated with lower levels of stress.
Carotenuto, Fasanaro, Molino, Sibilio, Saturnino, Traini & Amenta, (2013) [16] The Psychological General Well-Being Index (PGWBI) for assessing the stress of seafarers on board merchant ships	Quantitative and cross-sectional design	Male seafarers (n = 162) which consist of (1 Argentine, 1 Bulgarian, 122 Indians, 37 Italians and 1 Romanian) on board of 7 tankers in a shipping company	Anxiety, depressed mood, positive well-being, self-control, general health, and vitality	The general value is 0.80 and the highest 0.92 (Grossi & compare, 2014) [39]	No theoretical basis	Questionnaire was sent on board together with instructions and extensive explanations on how to administer it between August 2012 and April 2013. Captains of the ships were trained to the questionnaire administration and used as a reference point in case any clarification was required from the single seafarers	Seafarers are exposed to stressful conditions, some inevitably related to their activity (noise, vibrations, interrupted sleep, etc.), and other more subjective (individual capacity to endure loneliness, attitude to resilience, etc.)
Rydstedt & Lundh (2010) [38] An Ocean of Stress? The relationship between psychosocial workload and mental strain among engine officers in the Swedish merchant fleet	Quantitative and cross-sectional design	Swedish Merchant Marine Officers’ Association which consists of a total of (n = 731) engine officers in the Swedish merchant fleet. The British comparison sample consisted of (n = 312) professional shore-based engineers	No dimension for PSS10 For JCQ, Demands, Work-related control, decision authority in the work situation and skill variety, work-related social support	Perceived Stress Scale, version 10 (PSS10) and Job Content Questionnaire (JCQ) The PSS10 value is 0.84 and the value for JCQ is 0.77	Lazarus original transactional model and Job Demand Control Model	A questionnaire comprising 129 items was distributed to all engine officers affiliated with the Swedish Merchant Marine Officers’ Association	The main source of the high stress among the engine officers does not seem to be the job content but may rather be understood from an interactional perspective, where conflicting requirements are directed towards the individual officer.
An, Liu, Sun, & Liu (2020) [45] Impact of Work–Family Conflict, Job Stress and Job Satisfaction on Seafarer Performance	Quantitative and cross-sectional design	A merchant ship seafarer (n = 337) consisting of officers, engineers, ratings or crew, and catering in the Yangshan Port, Shanghai, China	No specific dimensions, just a list of work-related stress items	Questionnaire survey adapted from the literature The value reported is 0.85	No theoretical basis	Data gained onboard through the questionnaire. Messages regarding research information were sent from the Port State Control (PSC) to the captains, requesting them to notify seafarers onboard finishing survey forms within the specified time.	Job stress has associated negatively with job performance where it can reduce the performance of the seafarers
Håvold (2015) [2] Stress from the bridge of offshore vessels: Examples from the North Sea	Quantitative and cross-sectional design	The sample consists of Norwegian bridge officers (n = 157) working in theNorth Sea on offshore vessels	No specific dimensions, just a list of work-related stress items	Questionnaire survey adapted from the literature The value reported is satisfactory reliability	No theoretical basis	Questionnaire was developed based on a review of the literature and went through the process of checking for reliability and validity. The questionnaire was produced only in the Norwegian language All 40 non-nominal items in the questionnaire	The research shown that age and the length of time do not have any influence on stress and it differs based on the occupation (Between first mate and other navigators).
Rengamani & Venkatraman (2015) [25] Study on the job satisfaction of seafarers while on stress predicament	Quantitative and cross-sectional design	Seafarers of Indian origin (n = 385) who were working at various levels/job categories on the deck side and the engine side of foreign-going merchant vessels. The total sample size considered for the study was 385.	No specific dimensions, just a list of work-related stress items	Questionnaire survey constructed for the study The value reported for each item ranges from 0.78 to 0.93	No theoretical basis	Questionnaire was sent to participants of the target population	The majority of stressors tend to be those associated with psychological and social issues that are related to both personal and work lives.
Rengamani & Murugan (2012) [17] A study on the factors influencing seafarers’ stress	Quantitative and cross-sectional design	Indian Seafarers (n = 385) from various levels/job categories on the deck and engine sides of foreign-going merchant vessels.	No specific dimensions, just a list of work-related stressors items	Questionnaire survey constructed for the study The value reported for each item ranges from 0.71 to 0.82	No theoretical basis	Questionnaire was sent to seafarers of the target population	Important stressors on board are long working days, heat in workplaces, separation from their family, time pressure/ hectic activities, and insufficient qualifications of subordinate crew members. The seafarers who have high stress level because of heat in shipboard operations had shorter job duration at sea. The stressors of heat and noise show that physical stressors on ships currently are still very important in spite of the increasing mechanization in seafaring.
Elo (1985) [46] Health and stress of seafarers	Quantitative and cross-sectional design (two phases of study)	Seafarers (n = 591) from the Finnish m merchant fleet.	No specific dimensions, just questions related to stress was used	Questionnaire survey that claimed to be used in another study The value is 0.72	No theoretical basis	Questionnaire was sent to seafarers while onboard the ship, twice in both of the official languages of Finland (Finnish and Swedish).	Stress results varied for different occupational groups, the engine crew reporting the most stress and found to have problems in connecting with the work environment (i.e., noise, heat).

**Table 4:** Theme of physical stress.

Theme: Physical Stress	Participants	Responses
Weather	1	*“We are in the ships exposed to weather that cannot be controlled. The weather sometimes could affect our work concentration and rest.”*
	2	*“Sometimes they are more stressed because of the uncontrolled weather during sailing.”*
	22	*“When we are onboard, which could come from changes in the weather, people really affect our motivation to work and concentrate.”*
	25	*“During our sailing, sometimes when we are concentrating and excited in working, it suddenly rains, and for that reason, the weather really spoiled us.”*
Type of ship Location	2	*“Seafarers usually work in different ships, so the severity of the stress might differ according to the type of ship and whether they used to work in an LNG ship for a certain contract. If the next contract is with the same type of ship, they might feel less stress because they are used to the environment in that type of ship.”*
	10	*“We, as seafarers, most of us will not stick to particular ships in working; there are ships which somehow have so much stress due to their management, stress because of co-workers. Every ship is different.”*
	14	*“Stress or not actually depends on our management; even the type of ship can influence our stress level; a certain ship like a merchant vessels is a demanding ship which could be stressful for some seafarers.”*
	21	*“Type of ship and the management affect seafarers’ stress level.”*
	1	*“If we got to work at the port, we actually like working near the land. The environment is easier to accept because they can still see people moving around. Compare this to those who go to sail in the middle of the ocean, far away from land, they can only see the sun, the moon and stars.”*
	7	*“Stress levels differ between those who have to work onboard and those who are at the port.”*
	17	*“For me, every location is stressful, at the port we are stressed with noise, but we are near land; if on the sea, we are stressed missing family because we are far away.”*
	22	*“Every location is stressful depending on how we see it.”*
	23	*“I think at the port is quite tiring, but we are near to land, still not far to escape compared to those who are in the middle of the ocean.”*
Noise	6	*“Another stress factor is noise, especially if our rest place is near the engine room, surely, sometimes we can’t rest well.”*
	13	*“Usually, we cannot sleep well because of noise.”*
	23	*“Sometimes just the noise can make us tired and stressed.”*
Confined space	9	*“Yes, stress sometimes feels like we are locked up because of restrictions in the ship, we cannot go anywhere, everything is on the ship, is a confined area.”*
	11	*“The ship is limited in space. Almost everything we have to share with other crews, sometimes we feel like we need our privacy.”*
	12	*“The confined space is stressful like anything, and everything needs to be done within the space. “*
	16	*“In the ship, a lot of things are limited and a bit stress and it is difficult to share many things with the other members.”*
	25	*“Confined space is another stress factor among seafarers.”*

**Table 5:** Theme of personal issues.

Themes: Personal Issues	Participants	Responses
Character of seafarers	1	*“The character of seafarers contributes to stress. The more outgoing the person, the more likely they are to have friends compared to those who are not, so if they do not have people to interact with, they might experience stress. Also, the personality of seafarers of an able-to-go tough life will affect how they work on board.”*
	15	*“I think the first thing in reducing the stress for seafarers, is their own self, their own character.”*
	17	*“A good seafarer, who enjoys working, yeah, with less stress, are those who have good character, they enjoy the nature of working as seafarers.”*
	21	*“Look at the senior seafarers, those with good character. Most of them rarely stress working onboard, they enjoy it.”*
Background of seafarers	8	*“When I interviewed junior seafarers who applied for jobs, when they said their background is from a rich family, I do not feel confident accepting them, because in the past, seafarers who came from this so-called rich background most of the time can’t retain a seafaring career because it is difficult facing stressful things in this career.”*
	23	*“A lot of seafarers who experience stress and have mental health issues during work, most cannot cope with the condition because they came from wealthy families.”*
	25	*“When the seafarers used to live in a condition of hardship, difficulty, usually they can adapt well to working as seafarers.”*
Social status	1	*“The one who is married might have more stress than the single one.”*
	8	*“If talking about stress, the most important reason is that they are far away from their family, they miss their family, especially the married ones.”*
	9	*“The married seafarers usually stress about if anything happens because the wife has to handle their children alone.”*
	14	*“The single one does not have a family; they might not have much stress compared to the married one. The married seafarers usually stress more because they miss their wife and children.”*
	15	*“It’s stressful when we have to be far away from our family, especially if they are married.”*
Passion	2	*“The most important thing to have when you work as a seafarer is passion. If they just work as work, they might not really enjoy being a seafarer.”*
	3	*“The most important thing for work is passion. If not, you cannot go on, you may not enjoy doing the work”.*
	10	*“If you don’t have passion, you will feel the work is a burden for you, and you will not happy doing the work.”*
	17	*“The old or senior seafarers, if you can ask them, most of them are very passionate, that is why the senior seafarers were rarely involved in over-stressing problems.”*
	21	*“If I can say something to those who have the intention to join seafaring, this work needs those who are passionate because the nature of the work is naturally stressful, being in the middle of ocean, far away from family, living in a confined space. If you came work without passion, you cannot survive.”*
Awareness	2	*“The seafarers were still unaware of the stress condition and could not identify if they have a stress-related problem.”*
	4	*“Sometimes they do not know how to handle it and where to reach when they have a problem, especially related to mental stress, and the level of readiness of the awareness of stress is still less among seafarers.”*
	7	*“Stress is something we can’t see although we can feel it. Some people do not know they are actually in a condition of stress, they are actually not aware maybe because they do not know it.”*
	8	*“Awareness of stress is important because we could tackle the problem earlier before it worsens.”*
	25	*“If the seafarers are always aware and check themselves, take action if needed, this problem of stress could be prevented at an earlier stage.”*
Experience	4	*“Not having much experience can also lead to stress, Not having much preparation, especially for the new workers, will be stressful because we might experience culture shock.”*
	6	*“Experience could be a factor of stress. Those who are newcomers will usually stress be facing this nature of work for the first few years. If they commit and have passion, after some experience they will get better.”*
	17	*“I think stress is normal for those who join this seafaring profession, and after having experiences in this working area, we may understand the work nature and could be better in handling our stress and problems.”*
	19	*“Gain as many experiences, slowly we can adapt and enjoy the work, the more experiences, the less the stress.”*

**Table 6:** Theme of social living onboard.

Theme: Social Living on Board	Participants	Responses
Interaction with people	1	*“In the old time, more people on the ship, if they have more friends there, the social living on board will be slightly easier. Now we have the technology to handle, so manpower is less with fewer people on board to interact with.”*
	20	*“It is important for seafarers on board to always interact among them to avoid feeling stressed.”*
	22	*“Seafarers onboard area like one family. They should be able to interact and share their problems. This will make them enjoy being there and help them solve the problems.”*
	23	*“The social interaction among the crew onboard plays an important role in reducing stress and avoid feeling lonely.”*
Fewer social activities	7	*“Seafarers’ life onboard nowadays compared to the old one. They do not have social activities onboard that could make them closer and also help them reduce the stress of working.”*
	9	*“It’s kind of boring onboard, especially after finish working of not knowing what to do.”*
	21	*“One of the ways that crew in the board can do to reduce their stress from working is to have more social activities among the crew members.”*
	25	*“We need to have activities onboard so we can distract ourselves from work routines.”*
Cannot escape when facing conflict	13	*“Conflict between crew will be problematic and stressful because we cannot avoid meeting them because we are on the same ship.”*
	23	*“Usually, when we are in conflict, the best way is to escape and build some gap. This cannot be done if we are onboard and sure this will be stressful.”*
	25	*“If we can, we will try our best to avoid any conflict, especially with our team members, because we cannot escape and run even for a while.”*

**Table 7:** Theme of technostress.

Themes: Technostress	Participants	Responses
Affects concentration	6	*“Technology like email, and WhatsApp have made us receive an overload of information of work-related things sometimes are tiring and stress.”*
	7	*“When everything is through technology, we forget sometimes we spend too much on the gadgets even for work purposes, which could affect our health.”*
	16	*“Technology helps to spread things easier, like through WhatsApp but sometimes it’s too much, and we can be stressed over it.”*
	18	*“We are happy we could contact our family with the technology but sometimes too in contact will make us less focused on work.”*
	19	*“Social media really disturb our concentration to do work, and its tiring and stressful.”*
Skills deficit	12	*“The machine thing will help us in certain things, but it may make us less skilled in certain thing.”*
	15	*“The current seafarers are skilled in technology, but if they ask to complete a certain task manually, sure they cannot do it well.”*
	17	*“The stressful thing about technology is we will not have skilful seafarers who can do work manually, let’s say if a machine does not work.”*
	22	*“Nowadays seafarers are easy to compare to us, but they are more stressful actually, let’s say when the machine thing cannot operate, they have to do certain skills manually and if they cannot perform well. It could be a problem.”*
	24	*“The problem with technology is, yes, to have less skilful seafarers who depend too much on machines.”*

**Table 8:** Theme of work factors.

Themes: Work Factors	Participants	Responses
No guidance from seniors	12	*“Seniors always say to us to learn by ourselves, but we sometimes do not know what we do is right or not, they don’t guide us properly.”*
	13	*“I think if we get proper guidance from the senior, we could do work fast with less stress.”*
	16	*“Yes, knowledge is important, but sometimes certain problem is beyond us knowledge, we need to seek advice from senior. There is a senior willing to help, there is also those who ignores us.”*
	24	*“Cooperation between junior and senior seafarers are important to ensure the work can be done better, senior who have more experience will help juniors who just came in.”*
Workload	3	*“The workload is high and sometimes we don’t get the proper rest.”*
	4	*“The workload also increases. Sometimes we have been given work or tasks out of our job scope.”*
	21	*“The thing is, top management increased our workload, I thought with technology manpower is reduced, and we will not have to stress of workload problem, but we need to do more other things like paperwork.”*
	22	*“High workload means high stress.”*
Treatment of the employer	17	*“I think there is unfair treatment from the top management that makes some seafarers stress and do not want to continue in their career.”*
	19	*“I remember I worked with this company, and very good treatment I got; sometimes a stressful task can be enjoyed because they help us and treat us better.”*
	21	*“When we make a complaint and tell our concern to the top management, it is disappointing somehow, they ignore our concerns and complaints.”*
	23	*“If the top management care for a need and welfare of seafarers, surely the stress things could be reduced among them.”*
Job security	5	*“Most of the jobs in seafaring are contract basis. They do not know is they will get another contract if they finish a certain contract.”*
	6	*“The major concern among seafarers is actually about job security, which affects their performance, we could stress thinking of not being secure for a certain job.”*
	7	*“Job security is always a problem among seafarers, it is normal if they feel stress working and no guarantee for it.”*
	22	*“Job security is an issue that needs to be settled for seafarers.”*
	24	*“Yes, when we are not secure for a job, we are stressed because we need money to survive and to feed our family.”*
Working hours	3	*“They did not follow the stated working hours and forced us to work at any time.”*
	4	*“Long working hours sometimes could be stressful.”*
	6	*“Although seafarers have standard working hours, we do not follow them. We work more and do not have enough rest time.”*
	9	*“Yes, long working hours are stressful.”*
	11	*“Because we cannot move anywhere, confined in a ship, sometimes we need to work after the stated working hours and sometimes bring us stress, we cannot escape from the ship.”*
	15	*“Top management always say only work during working hours, but in reality, when we are on the ship, we work more than that.”*
Wages	1	*“Many seafarers are unsatisfied with the wage scale and all that.”*
	16	*“Seafarers, most of them are not satisfied with the wages scale.”*
	18	*“The problem of wages needs to be settled, which can reduce stress among seafarers.”*
	20	*“Wages always took time to be paid. Sometimes we need the money urgently.”*
	21	*“Earning money is the main thing when we are working, if it’s a problem for sure we will feel stressed.”*

**Table 9:** Theme of COVID-19 Pandemic.

Themes: COVID-19 Pandemic	Participants	Responses
Travel restriction	8	*“The travel restriction during covid19 is stressful. At the beginning, we are stuck, and missing our family.”*
	9	*“I still remember when the lockdown happened, we could not go anywhere. It was restricted and so much stress during the time.”*
	10	*“Everybody was stressed when we are restricted from going anywhere.”*
	14	*“Yes, the pandemic was stressful, especially during the early lockdown. We were somehow stuck did not know what to do, and could not go anywhere.”*
	21	*“It was stressful because we were stuck, could not travel back to meet family for months.”*
	24	*“The COVID-19 was historical and stressful, cannot go anywhere, and we were separate from family and could not do anything.”*
	25	*“When the Prime Minister announced the lockdown and travel restriction, we were shocked and went through a very hard time.”*
Getting unpaid	2	*“This COVID-19 is really stressful, especially when you don’t get paid.”*
	3	*“One problem for seafarers during this pandemic is they did not get their pay because they were stuck and could not sign in.”*
	4	*“Another effect of this COVID-19 is if we are stuck at home, we cannot go to work, and will not get paid.”*
	6	*“When there is no sign in sign off, we cannot work, and our money is reducing. That was very stressful”*
	22	*“The travel restriction during the pandemic, especially for the first few months, was really stressful for us, we cannot go to work and get money for our family.”*
	25	*“I feel so much stress because I had no money to give to the family and was stuck at home.”*
Uncertainty	1	*“Because of this pandemic, seafarers now do not know whether they can secure a job or not.”*
	20	*“So many uncertainties thinking of our work.”*
	21	*“During the MCO, we were so much worry and stress because everything was uncertain, start with not sure when to sign in sign off to our work contract, whether we can continue working or not.”*
Double standard quarantine	4	*“Some people are not given fair period for the quarantine. There are people who can just quarantine for three days and some need to quarantine for fourteen days.”*
	7	*“There is unfairness in the quarantine period for certain people they do not follow quarantine period of fourteen days.”*
	8	*“We have to pay for quarantine ourselves, which was stressful.”*
	9	*“It was such a stressful experience when we had to quarantine for 14 days, and there are some people have to quarantine less than that.”*
Standard Operating Procedure (SOP) restrictions	6	*“Standard operating procedure (SOP) made us stressed, many things to do and sometimes tiring.”*
	7	*“SOP comes with many rules need to follow, its stressful.”*
	11	*“I remember during the covid19 phase, we are not only stressed because cannot go anywhere, no income, but we stress because of SOP things.”*
	12	*“I feel somehow the SOP is nonsense sometimes and tiring.”*

## Data Availability

Not applicable.
